# Fundamental Molecules and Mechanisms for Forming and Maintaining Neuromuscular Synapses

**DOI:** 10.3390/ijms19020490

**Published:** 2018-02-06

**Authors:** Steven J. Burden, Maartje G. Huijbers, Leonor Remedio

**Affiliations:** 1 Helen L. and Martin S. Kimmel Center for Biology and Medicine at the Skirball Institute of Biomolecular Medicine, New York University Medical School, 540 First Avenue, New York, NY 10011, USA; lm3256@columbia.edu; 2 Departments of Neurology and Human Genetics, Leiden University Medical Center, 2333 ZA Leiden, The Netherlands; M.G.M.Huijbers@lumc.nl; 3 Zuckerman Mind Brain Behavior Institute, Columbia University, 3227 Broadway, New York, NY 10027, USA

**Keywords:** Agrin, Lrp4, MuSK, Dok-7, Rapsyn, Acetylcholine receptors, motor neurons, ALS, congenital myasthenia, myasthenia gravis

## Abstract

The neuromuscular synapse is a relatively large synapse with hundreds of active zones in presynaptic motor nerve terminals and more than ten million acetylcholine receptors (AChRs) in the postsynaptic membrane. The enrichment of proteins in presynaptic and postsynaptic membranes ensures a rapid, robust, and reliable synaptic transmission. Over fifty years ago, classic studies of the neuromuscular synapse led to a comprehensive understanding of how a synapse looks and works, but these landmark studies did not reveal the molecular mechanisms responsible for building and maintaining a synapse. During the past two-dozen years, the critical molecular players, responsible for assembling the specialized postsynaptic membrane and regulating nerve terminal differentiation, have begun to be identified and their mechanism of action better understood. Here, we describe and discuss five of these key molecular players, paying heed to their discovery as well as describing their currently understood mechanisms of action. In addition, we discuss the important gaps that remain to better understand how these proteins act to control synaptic differentiation and maintenance.

## 1. Rock Bottom Synaptic Transmission

**Figure 1:**
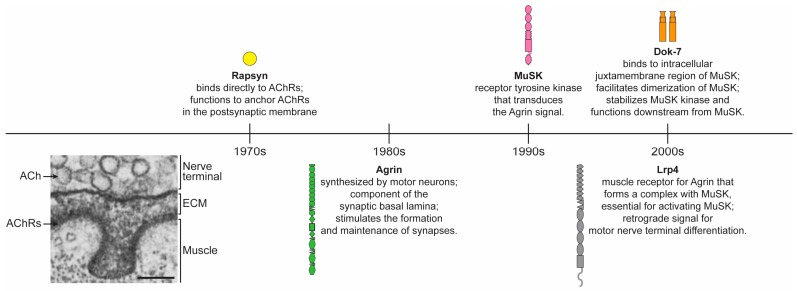
Timeline for the discovery of key molecular players at the neuromuscular synapse. The peripheral membrane protein Rapsyn binds to the intracellular region of AChRs and anchors AChRs and other muscle proteins in the postsynaptic membrane. Later studies identified Agrin as a critical motor neuron-derived signal that is deposited into the synaptic lamina and stimulates the formation and maintenance of neuromuscular synapses, including the clustering of AChRs. Shortly thereafter, MuSK was identified, shown to mediate the response to Agrin, and found to be critical for both postsynaptic and presynaptic differentiation. More than a decade elapsed before the Agrin receptor was identified as Lrp4. Lrp4 functions not only to bind Agrin, promoting association between Lrp4 and MuSK and stimulating muscle differentiation, but also acts in turn as a retrograde signal for presynaptic differentiation. During this same time period, Dok-7 was identified and found to bind to tyrosine phosphorylated MuSK, stabilize MuSK in an active tyrosine-phosphorylated state and function downstream from MuSK. ACh, Acetylcholine. AChRs, Acetylcholine Receptors. ECM, Extracellular matrix. MuSK, muscle specific kinase. Lrp4, LDL receptor related protein 4. Dok-7, docking protein 7. Bar, 100 nm.

Studies by Katz and colleagues established the fundamental mechanisms for synaptic transmission [1]. Following propagation of an action potential into the nerve terminal, an influx of calcium stimulates synaptic vesicles, containing ACh, to fuse with the presynaptic membrane, releasing ACh into the synaptic cleft. ACh binds to densely clustered AChRs in the muscle membrane, ensuring for rapid, robust and reliable synaptic transmission. The ligand-gated opening of AChR channels causes focal depolarization of the muscle, which initiates a muscle action potential that triggers muscle contraction. Following the studies by Katz and colleagues, critical molecules for building the neuromuscular synapse and concentrating AChRs in the postsynaptic membrane were identified. Five of these key proteins are discussed here (Figure 1).

## 2. Fundamental Proteins of the Neuromuscular Synapse

### 2.1. Agrin

Although it had long been appreciated that motor neuron-derived factors played an important role in postsynaptic differentiation, including clustering of AChRs, the critical neuronal factors remained undiscovered through the 1970s. In the 1970s, several labs pursued the characterization and purification of soluble factors that were capable of stimulating clustering of AChRs in cultured muscle cells, reasoning that these factors either represented or mimicked the signal(s) that motor axons supplied to muscle to stimulate clustering of AChRs and postsynaptic differentiation at synapses. U.J. McMahan, who had been studying regeneration of neuromuscular synapses, took a different view. Because the synaptic extracellular matrix was interposed between the nerve terminal membrane and the postsynaptic muscle membrane at adult synapses [1], and because this extracellular matrix became apparent at synapses as they form during development [2], McMahan proposed that key factors for forming and maintaining synaptic differentiation may be located within the synaptic extracellular matrix, or basal lamina. In this view, the key differentiation factors were not membrane-bound; instead, the factor(s) were hypothesized to be released by motor axons or muscle and to be stably maintained in the synaptic extracellular matrix, where they would act to stimulate presynaptic and postsynaptic differentiation. Over the next several years, McMahan’s seminal experiments to test these ideas transformed thinking not only about synapse formation but also about the role of the extracellular matrix as a stable repository of signals instructive for cellular differentiation rather than simply providing structural support to regulate cell shape.

In the first set of experiments, adult motor axons and myofibers were damaged, leading to degeneration of distal motor axons and myofibers, but leaving the muscle fiber basal lamina, including the synaptic basal lamina, intact. Despite the absence of nerve terminals and muscle, original synaptic sites could be identified, due to the preservation of unique structural features of the synaptic basal lamina and the retention of acetylcholinesterase (AChE) in the synaptic basal lamina [3]. After a short period of time, motor axons and myofibers regenerated, and regenerated synapses formed nearly exclusively at original synaptic sites [4]. In the next set of experiments, nerve and muscle were damaged, but muscle regeneration was prevented. Despite the absence of myofibers, regenerating motor axons returned selectively to the original synaptic sites, where they differentiated and formed active zones [5]. These studies indicated that factors, which were stably maintained within the synaptic basal lamina, could control the differentiation of motor nerve terminals. In the third set of experiments, following damage to nerve and muscle, motor axon regenerated was prevented. Despite the absence of motor axons, regenerated myofibers became specialized at original synaptic sites, where AChRs clustered and postjunctional folds reformed [6]. These studies indicated that key factors for postsynaptic differentiation were likewise stably maintained in the synaptic basal lamina and set the stage for purification of these synapse-inducing factors, which were presumed to be synthesized by nerve and muscle and deposited in the synaptic basal lamina. These factors were hypothesized to function not only during regeneration but also to act during development to induce synaptic differentiation during synapse formation [6].

Synapses in the electric organ of *Torpedo californica* look and function like neuromuscular synapses. Importantly, because one entire surface of the electrocyte is studded with nerve terminals, whereas only 0.01% of the myofiber membrane is contacted by motor nerve terminals, the enrichment of synaptic proteins is several thousand-fold greater in the electric organ than in muscle, simplifying purification. Moreover, it is simple to begin with several kg of electric organ tissue, whereas it would be difficult to begin with more than several grams of skeletal muscle tissue. For this reason, the electric organ had been the source for purification of key components of the neuromuscular junction, including AChRs, AChE, and Rapsyn (see below). Therefore, it seemed reasonable that synaptic basal lamina components, responsible for synaptic differentiation, would be contained and enriched in the electric organ as well. Because it was far easier to assay for clustering of AChRs in cultured muscle cells than formation of active zones in nerve terminals, the studies focused on the extracellular matrix factor(s) responsible for clustering AChRs. Fractionation and purification of an activity from the electric organ that stimulated clustering of AChRs in cultured muscle cells led to the identification of a protein dubbed Agrin [7,8]. Subsequent studies demonstrated that Agrin is present in the synaptic basal lamina at neuromuscular synapses, as well as at synapses in the electric organ, and plays a critical role in the formation and maintenance of neuromuscular synapses [9,10,11].

The *Agrin* gene encodes a large, ~200 kDa protein with three laminin-G-like domains and four EGF-like domains contained in the carboxy-terminal half of the protein [11]. The N-terminal region of Agrin contains a laminin-binding motif, responsible for tethering Agrin to the synaptic basal lamina. The *Agrin* gene is expressed in multiple cell types, but neurons express isoforms, containing 8, 11 or 19 amino acids near the carboxy-terminus, generated by alternative splicing, which are critical for stimulating AChR clustering [12,13,14]. A 50 kDa carboxy-terminal fragment form of neuronal Agrin, containing two EGF-like domains and two laminin-G-like domains, is as active as full-length Agrin when presented to cultured myotubes [15], whereas the N-terminal laminin-binding motif is essential for Agrin to function in vivo.

Agrin is also expressed in the central nervous system (CNS). Here, Agrin is a type II transmembrane protein rather than an extracellular matrix protein [16]. The CNS forms of Agrin are generated by usage of a different promoter, which dictates a different pattern of alternative splicing, excluding the laminin-binding region and instead encoding a transmembrane segment. The role of Agrin in the CNS is poorly understood.

### 2.2. MuSK

The quest for an Agrin receptor began shortly after the discovery of Agrin. A false lead arose from several studies reporting that α -dystroglycan was the functional receptor for Agrin [17,18,19,20]. However, it soon became clear that although α-dystroglycan can bind neuronal, as well non-neuronal forms of Agrin, α-dystroglycan was not the functional receptor for Agrin [21,22,23]. In the 1980s, it became appreciated that receptor tyrosine kinases (RTKs) often mediate the response to signals that control cellular differentiation. It therefore seemed plausible that the Agrin receptor might be an RTK. Consistent with this idea, antibodies to phosphotyrosine showed strong staining at synapses in muscle and in the electric organ [24]. To identify RTKs that might participate in synaptic differentiation, degenerate primers were used to amplify RTKs from *Torpedo* electric organ, reasoning that a synaptic RTK would be unusually abundant in this synapse-enriched tissue. This assay identified a single, novel RTK that was particularly abundant in the electric organ and also expressed in muscle [25]. This kinase, dubbed *Torpedo* RTK, is a single pass transmembrane protein with a kinase domain similar to that in Trk neurotrophin receptors and an extracellular region with multiple Ig-like domains and a kringle domain. Subsequent studies pointed to the additional presence of a cysteine-rich, Frizzled-like domain in the extracellular region [26,27]. In other respects, the amino acid sequence of the electric organ/muscle RTK appeared unlike any known membrane receptor. Subsequent studies showed that the mammalian homologue, dubbed MuSK, is enriched at mammalian neuromuscular synapses and expressed by skeletal muscle but not by motor neurons [28]. Mammalian MuSK contains all of the domains present in the *Torpedo* RTK, but mammalian MuSK, unlike fish, amphibian and avian MuSK, lacks the kringle-like domain [29]. Alternative splicing of *MuSK* RNA can generate multiple MuSK isoforms, but the functional significance of these isoforms is not known. Subsequent studies showed that MuSK is also expressed in the CNS [30,31], but the function of CNS MuSK has not received due attention. MuSK has also been reported to be expressed by additional cell types [32,33], but the function of MuSK in these other cell types has likewise not been studied. MuSK, as well as neuronal Agrin and Lrp4 (see below) are expressed in hepatocellular carcinoma (HCC) cell lines [34]. Activation of MuSK by Agrin enhances cell proliferation, promotes migration, and stimulates tumor growth. If this HCC tumor model reflects HCC in humans, these findings would suggest that MuSK signaling contributes to tumorigenesis.

Mice lacking MuSK die at birth from respiratory failure, due to a failure to form neuromuscular synapses [35]. In the absence of MuSK all features of presynaptic and postsynaptic differentiation fail to develop [35], indicating that MuSK plays an essential role in synapse formation. AChRs, as well as other proteins that are normally enriched at synapses, are expressed by muscle but no longer enriched at synapses in *MuSK* mutant mice, consistent with the idea that Agrin and MuSK function in the same signaling pathway. Moreover, synapse-specific transcription also requires MuSK, as ‘synaptic’ genes, which are normally transcribed preferentially by synaptic myofiber nuclei, are expressed similarly in all nuclei throughout muscle of *MuSK* mutant mice [35,36,37].

Agrin stimulates tyrosine phosphorylation of MuSK in cultured myotubes, demonstrating that Agrin can activate MuSK [38]. However, ligands that bind directly to RTKs stimulate RTK phosphorylation within a minute, whereas over five minutes are required for Agrin to stimulate full phosphorylation of MuSK [38], suggesting that additional steps intercede between Agrin-binding and stimulation of MuSK kinase activity. Indeed, Agrin binds MuSK only very weakly [39], and fails to stimulate MuSK phosphorylation in transfected non-muscle cells expressing MuSK [38]. Together, these studies indicated that although MuSK transduces the Agrin signal, MuSK is not itself an Agrin receptor.

Surprisingly little is known about the mechanisms by which MuSK mediates the clustering and anchoring of postsynaptic proteins as well as synapse-specific transcription. Once the activation loop tyrosine residues in the MuSK kinase domain, as well as a single tyrosine residue in the juxtamembrane region, are phosphorylated, MuSK becomes an active kinase [40,41,42]. Phosphorylation of the tyrosine in the MuSK juxtamembrane region leads to recruitment of a key adapter protein, downstream of kinase-7 (Dok-7; see below), which is thought to have two essential roles: (1) to maintain MuSK tyrosine phosphorylation, necessary to sustain MuSK kinase activity and (2) to function as a substrate for MuSK kinase, promoting recruitment of two further adapters, Crk and Crk-L, to two tyrosine phosphorylated motifs in the carboxy-terminal region of Dok-7 (see below).

There is good evidence that actin remodeling participates in clustering of AChRs. Stimulation of MuSK by Agrin activates Rac and Rho, and they play an important role in the formation of micro- and macroclusters of AChRs that form in cultured myotubes [43,44]. Moreover, actin polymerization and regulators of F-actin assembly, such as Cortactin, together with an Arp2/3 complex, are enriched at AChR clusters in cultured muscle cells [45,46]. How these pathways are activated by MuSK and how actin and actin regulators act to assist in the anchoring of postsynaptic proteins is not understood.

Additional proteins, including Magi-1c, a protein with a PDZ-binding domain, and two different E3 ubiquitin ligases, one containing a PDZ-binding domain, can bind to the intracellular region of MuSK [47,48], but the physiological significance of these interactions is not understood. The collagen Q (ColQ) subunit of AChE can bind to the extracellular region of MuSK [49], raising the possibility that this association, together with the strong binding between ColQ and Perlecan, an extracellular matrix protein, may assist in anchoring the ColQ form of AChE to the synaptic basal lamina.

MuSK is required to maintain as well as to form neuromuscular synapses, since reducing MuSK expression by RNAi or by conditional gene inactivation in adult myofibers leads to synaptic disassembly [50,51]. Moreover, rabbits and mice immunized with MuSK show reduced AChR surface expression and impaired synaptic transmission, which lead to muscle weakness, akin to deficits found in MuSK myasthenia gravis (MG) (see below) [52,53]. Consistent with the idea that MuSK functions to maintain as well as to form neuromuscular synapses, approximately 15% of patients with MG have autoantibodies to MuSK, and passive transfer of these antibodies disrupts the mouse neuromuscular junction and induces muscle weakness [54,55,56].

In addition to postsynaptic defects, motor axons fail to stop and differentiate in *MuSK* mutant mice, indicating that MuSK not only plays a muscle autonomous role in postsynaptic differentiation but also has a non-autonomous role in presynaptic differentiation. How MuSK is thought to participate in presynaptic differentiation will be discussed below (see Section 2.3).

### 2.3. Lrp4

The discovery of MuSK revealed much about the mechanisms by which Agrin stimulates postsynaptic differentiation. However, because Agrin does not bind directly to MuSK [38], it remained unclear how Agrin stimulated MuSK and initiated the MuSK-mediated transduction pathway. Once it was established that MuSK played a critical role downstream from Agrin, a dozen years passed while multiple labs hunted for the Agrin receptor, which presumably had a key role in activating MuSK. 

A recessive screen for genes required for early steps in mouse development identified two mutant alleles of *Lrp4* [57]. These *Lrp4* null mutants had the expected digit defects, due to a loss of Lrp4 function, but in addition, the *Lrp4* mutant mice died at birth due to respiratory failure [57]. Further characterization revealed deficits in neuromuscular synapse formation that resembled the abnormalities found in *Agrin* and *MuSK* mutant mice, raising the possibility that Lrp4 functioned in the Agrin/MuSK signaling pathway [57]. Consistent with this idea, *Lrp4* RNA is expressed in the central, synaptic region of muscle, like *MuSK* RNA [58], but not in developing motor neurons [57].

Subsequent studies demonstrated that Lrp4 is the functional muscle receptor for neuronal Agrin [39,59]. These studies showed that Lrp4 binds neuronal Agrin, forms a complex with MuSK and functions as a ligand to stimulate MuSK phosphorylation [39,59,60,61].

Lrp4 is a single-pass transmembrane protein composed of eight low-density lipoprotein receptor domain class A (LDLa) repeats, two epidermal growth factor (EGF)-like domains, and four beta-propeller (BP) domains, each of which is melded together with an EGF-like domain [29]. Neuronal Agrin binds to the first BP domain, although the last few LDLa repeats contribute to Agrin-binding [60,61]. The third BP domain has a critical role in binding of Lrp4 to MuSK [60,62,63]. Association between Lrp4 and MuSK, which is enhanced by binding of Agrin to Lrp4, is mediated, at least in part, by the first Ig-like domain in MuSK [60]. Thus, the extracellular region of Lrp4 contains all of the motifs necessary for Lrp4 to bind Agrin and associate with MuSK [60,64]. The intracellular region of MuSK is not required for neuromuscular synapse formation but has an important role for Lrp4 to regulate digit formation [64,65,66].

Consistent with biochemical studies, which identified domains in Lrp4 and MuSK that mediate their association, pathogenic autoantibodies to MuSK, which cause MG, bind the first Ig-like domain in MuSK, thereby inhibiting binding between Lrp4 and MuSK and reducing MuSK tyrosine phosphorylation [67,68]. Moreover, although certain mutations in human *Lrp4* cause Cenani-Lenz syndrome [69,70], characterized by bone malformations, other mutations in *Lrp4*, notably in one face of the third BP domain, impair binding to MuSK and cause congenital myasthenia [62,63].

Lrp4, like other postsynaptic proteins, becomes concentrated and anchored in the postsynaptic membrane as a consequence of MuSK signaling. As such, Lrp4 functions both upstream and downstream from MuSK. Once clustered by MuSK signaling, Lrp4 functions as a retrograde signal for presynaptic differentiation (see below), as Lrp4 is necessary and sufficient to induce differentiation of motor nerve terminals [71]. This third function of Lrp4, in addition to its function as an Agrin receptor and a MuSK ligand, is mediated by the LDLa repeats in Lrp4 [71]. The receptor for Lrp4, which functions in motor neurons to control presynaptic differentiation, has yet to be identified.

Lrp4, like Agrin and MuSK, is expressed in the CNS. Although the roles for Agrin and MuSK in the CNS are not well understood, several studies have demonstrated that Lrp4 has an important role in the development of CNS synapses [72,73,74,75]. How Lrp4 functions to control CNS development is not understood. Although one study reported that the alpha3 Na/K ATPase is the CNS receptor for Agrin [76], Lrp4 may function in the CNS, like in muscle, as the receptor for Agrin. In addition, because Lrp4 can stimulate presynaptic differentiation in cultured cortical neurons [77], it remains possible that mechanisms shared with neuromuscular synapses underlie, at least in part, the function of Lrp4 in the CNS.

### 2.4. Dok-7

Studies, which sought to identify regions of MuSK that are important for MuSK function, showed that a phosphotyrosine-binding (PTB) site in the MuSK juxtamembrane region plays a critical role in MuSK-dependent signaling [40,41]. These studies showed that muscle cells, which expressed MuSK with mutations in the core motif (NPXY) of the PTB site, which either substituted phenylalanine for tyrosine and prevented phosphorylation of the PTB site or modified other core residues in the PTB site to block recruitment of PTB domain-containing proteins, failed to cluster AChRs in response to Agrin. These findings suggested that tyrosine phosphorylation of the PTB site led to recruitment of a PTB domain-containing protein that was essential for signaling downstream from MuSK. Surprisingly, these mutations in the PTB site also prevented Agrin from stimulating phosphorylation of the three activation loop tyrosine residues in MuSK [41]. Because mutation of the PTB site blocked MuSK tyrosine phosphorylation in muscle cells, but not in vitro [41], the PTB site did not appear to have an intrinsic, intramolecular role in regulating MuSK tyrosine kinase activity, as described for the PTB site in EphB receptors [78]. Instead, these studies suggested that recruitment of a PTB domain-containing protein to tyrosine phosphorylated MuSK in muscle was necessary to stabilize MuSK tyrosine phosphorylation, which was critical for MuSK to stimulate AChR clustering and postsynaptic differentiation. The PTB site in MuSK had the canonical NPXY motif, but the sequence N-terminal to the core NPXY motif was dissimilar to PTB sites for well-known PTB domain-containing proteins, suggesting that MuSK recruited a novel PTB domain-containing protein.

The Yamanashi group had studied PTB domain-containing proteins that were members of the Dok family, and they searched databases for additional, unannotated Dok proteins. They identified a new family member, Dok-7, which is selectively expressed in skeletal and heart muscle, and showed that Dok-7 binds the tyrosine phosphorylated PTB site in MuSK [79]. In a single, comprehensive and elegant study, Okada et al. [79] not only identified Dok-7, but also demonstrated that Dok-7: (1) binds the PTB site in MuSK; (2) is recruited to MuSK following Agrin-stimulated MuSK tyrosine phosphorylation; (3) is concentrated at neuromuscular synapses in vivo; and (4) is essential for neuromuscular synapse formation in vivo.

Further studies provided insight into how Dok-7 works. Dok-7 is a 55 kDA protein and a member of the Dok adaptor protein family that consist of a N-terminal pleckstrin-homology (PH) domain, a PTB domain and Src homology 2 (SH2) domain target motifs in the carboxy-terminal region [80]. The PH/PTB domains in Dok-7 mediate binding to the tyrosine phosphorylated juxtamembrane region of MuSK [79,81]. In addition, the PH/PTB domains of Dok-7 mediate homodimerization of Dok-7 [82].

The Dok-7 carboxy-terminal region contains two tyrosine residues, Y396 and Y406, that are phosphorylated following recruitment of Dok-7 to tyrosine phosphorylated MuSK [81,83]. The sequences surrounding these tyrosine residues conform to SH2-binding sites, and once phosphorylated, they recruit Crk and Crk-L, adapter proteins containing SH2 and SH3 domains [81,83]. A common and simple way to assess Dok-7 function is to introduce wild-type or mutant forms of Dok-7 into muscle or non-muscle cells by transfection. Over-expression of Dok-7 in cultured muscle cells stimulates MuSK tyrosine phosphorylation and AChR clustering, even in the absence of Agrin stimulation. AChRs fail to cluster in muscle cells transfected with mutant forms of Dok-7 (Dok-7 396F; 406F), which cannot be tyrosine phosphorylated and recruit Crk/Crk-L, suggesting that recruitment of Crk/Crk-L plays an important role in clustering AChRs [81,83]. However, because Dok-7 overexpression leads to MuSK activation and AChR clustering, even in the absence of Agrin stimulation, these studies should be interpreted with caution as they may not reflect the mechanisms by which Dok-7 normally acts in vivo. Muscle-conditional inactivation of Crk and Crk-L leads to synaptic defects [83], consistent with the findings from Dok-7 over-expression in cultured muscle cells, but these defects could be due to a loss of binding between Crk/Crk-L and Dok-7 or a loss of other functions for Crk/Crk-L in muscle.

Agrin fails to stimulate MuSK tyrosine phosphorylation in muscle cells lacking Dok-7 [84]. These findings, which are consistent with prior studies showing that the MuSK PTB site is required for MuSK phosphorylation, indicate that binding between Dok-7 and tyrosine phosphorylated MuSK stabilizes MuSK tyrosine phosphorylation [41]. Structural studies, which show that Dok-7 functions as a dimer to promote MuSK dimerization and trans-phosphorylation, support this idea and provide a mechanistic understanding to explain how Dok-7 might participate to stabilize MuSK phosphorylation [82]. Thus, Dok-7 appears to function, at least in part, as an inside-out ligand to stabilize and promote MuSK tyrosine phosphorylation.

Although the carboxy-terminal region of Dok-7 is not essential for Dok-7 to stimulate MuSK tyrosine phosphorylation, the carboxy-terminal region of Dok-7 is required to stimulate maximal MuSK tyrosine phosphorylation in vitro [85]. The mechanism by which the carboxy-terminal region of Dok-7 contributes to stimulate MuSK phosphorylation is not understood. The carboxy-terminal region, which is likely unstructured, may stabilize a Dok-7 dimer and thereby facilitate MuSK phosphorylation. The role of the Dok-7 carboxy-terminal region in stimulating MuSK phosphorylation is only evident in myotubes and not in heterologous cells or myoblasts. These data suggest that Dok-7-dependent MuSK activation in myotubes is regulated by a cell type-specific mechanism that is not yet understood [41,85].

Approximately, 10–15% of patients diagnosed with congenital myasthenia carry mutations in *Dok-7* [86,87,88]. Certain disease-causing mutations in *Dok-7* impair the ability of Dok-7 to bind MuSK, while other mutations reduce Dok-7 dimerization [89], each leading to a reduction in AChR clustering [87]. The most common mutation is a four-bp duplication (1124_1127 TGCC), which leads to a frame-shift and premature truncation of Dok-7, resulting in a loss of the two carboxy-terminal tyrosine residues [86,90]. Although neuromuscular synapses are structurally and functionally impaired in patients who are homozygous for this mutation, the mutation does not cause lethal respiratory failure. As such, this mutation, which eliminates the two carboxy-terminal tyrosine residues, is not a loss of function mutation, indicating that the PH/PTB domains of MuSK, which mediate Dok-7 dimerization and promote MuSK tyrosine phosphorylation are alone able to stimulate, albeit incomplete, synaptic differentiation. These findings suggest that MuSK phosphorylation is ‘sensed’ by additional proteins, which bind either to MuSK or to the PH/PTB domains in Dok-7 and participate in signaling downstream from MuSK.

Like *MuSK* mRNA, *Dok-7* mRNA is enriched in the central region of developing muscle at E14.5 [84]. This enrichment of *Dok-7* RNA, and presumably Dok-7 protein, likely contribute to MuSK activation and muscle pre-patterning. However, this enrichment of *Dok-7* mRNA in the central region of muscle is transient, and by E18.5, *Dok-7* mRNA, unlike MuSK and AChR mRNAs, is uniformly expressed in muscle [84], indicating that the *Dok-7* gene is not a target of MuSK signaling. The enrichment of *Dok-7* mRNA at E14.5 may be a consequence of the pattern of muscle growth: because myoblasts fuse at the growing ends of the myotube, genes that are activated following myoblast fusion are expressed for a longer period of time in the central region of muscle, allowing for their transcripts to accumulate.

Dok-7 can shuttle between the nucleus and the cytoplasm [81]. The PH-domain of Dok-7 confers nuclear import, whereas the carboxy-terminal region contains a nuclear export signal (NES), which ensures that Dok-7 is translocated to the cytoplasm. Dok-7 is not known to have a nuclear function. Mutation of the nuclear export signal leads to accumulation of Dok-7 in the nucleus, which diminishes MuSK tyrosine phosphorylation and AChR clustering [81]. It remains possible that mutations in the carboxy-terminal region of human Dok-7, which truncate Dok-7 and lead to a loss of the NES, cause congenital myasthenia (see below) by impairing Dok-7 translocation to the cytoplasm.

### 2.5. Rapsyn

Rapsyn is a peripheral membrane protein that binds directly to skeletal muscle AChRs. Only two other proteins, Src-family kinases and adenomatous coli (APC) polyposis, have been reported to bind directly to AChRs. Src-family kinases have a role in stabilizing AChRs [91], whereas the role of APC in synaptic differentiation is poorly understood. In contrast, Rapsyn plays an essential role in anchoring postsynaptic proteins, including AChRs, in the postsynaptic membrane. Although Rapsyn was identified as an AChR-associated protein in the 1970s [92], long before the discovery of Agrin, Lrp4, MuSK, or Dok-7 (Figure 1), surprisingly little is known about how Rapsyn functions to anchor postsynaptic proteins. Difficulties in isolating purified, soluble, well-folded Rapsyn likely underlie the absence of structural information about Rapsyn. Moreover, Rapsyn-associated proteins likely play an important role in providing a scaffold for anchoring postsynaptic proteins, but very few Rapsyn-associated proteins have been identified, and none have been shown to play a key role in anchoring AChRs or other postsynaptic proteins (Figure 1).

Rapsyn co-isolates with AChR-rich postsynaptic membranes and was initially thought to be a subunit of the AChR and to contribute to the ion channel [92]. Biochemical as well as reconstitution experiments from bona fide *AChR* subunit mRNAs demonstrated that Rapsyn is neither an integral nor a necessary subunit of the AChR, but instead binds to the main intracellular loop, between the third and fourth transmembrane segments, of AChR subunits [93,94]. Rapsyn can bind each of the four AChR subunits [95]. In addition, following Agrin stimulation, the AChR β subunit becomes tyrosine phosphorylated [24], leading to recruitment of Rapsyn to the tyrosine phosphorylated AChR β subunit and therefore additional Rapsyn to the AChR complex [96].

Rapsyn is a myristolylated 43 kd protein with three notable domains: seven tetratrichopeptide (TPR) repeats, which constitute the N-terminal half of the protein; a coiled-coil region; and a C-terminal RING finger domain [97,98,99]. The coiled-coil region binds AChR subunits and is also required for Rapsyn to bind alpha-actinin [100]. The RING finger domain possesses catalytic activity that promotes neddylation of AChR subunits [101], and in addition, the RING finger domain binds dystroglycan [99]. The TPR repeats are thought to mediate oligomerization of Rapsyn [98], and in addition, they bind Macf1, a scaffolding protein that contains spectrin-like repeats as well as binding sites for microtubules and actin [102]. 

The thirty-four amino acid TPR repeat adopts a helix-turn-helix fold, and in proteins with multiple TPR repeats, the TPR repeats form a super-helical structure with the concave surface usually involved in ligand-binding. The shape of the super-helical structure depends upon residues that are interposed between adjacent TPR repeats. TPR repeats can mediate dimerization and oligomerization of TPR domain-containing proteins as well as binding to ligands [103,104]. In PEX5, the seven TPR repeats fold to form two subdomains, composed of TPR repeats 1–3 and 5–7, separated by a flexible TPR repeated 4. The cavity formed between the two subdomains binds cargo for import into the peroxisome [105]. Rapsyn also contains seven TPR repeats, so it is interesting to speculate that Rapsyn may adopt a structure akin to PEX5 and bind ligands in an analogous manner. However, instead of forming a cavity, the TPR repeats in Rapsyn may adopt a more extended and open configuration, promoting association between TPR repeats in adjacent Rapsyn molecules, thereby contributing to Rapsyn oligomerization. Knowledge of the structure of Rapsyn would be an important step in to understanding how Rapsyn functions.

In the absence of Rapsyn, AChRs fail to cluster and mice die perinatally [106]. Unlike Agrin, Lrp4, MuSK, and Dok-7, Rapsyn functions exclusively in the post-translational anchoring pathway and not in the synapse-specific transcription pathway. As such, in the absence of Rapsyn, like in wild-type mice. *AChR* and other ‘synaptic’ transcripts are enriched in the synaptic region [106]. Synaptic extracellular matrix proteins, such as Laminin β2 (S-laminin), which are secreted and not anchored in the membrane, mark ‘synaptic’ sites in *Rapsyn* mutant mice. AChRs are enriched in the central region of *Rapsyn* mutant muscle, due to synapse-specific transcription, but neither AChRs nor other postsynaptic membrane proteins are anchored in the ‘synaptic’ membrane, marked by S-laminin [106]. MuSK appears unique among transmembrane proteins, as MuSK is concentrated at defunct ‘synaptic’ sites in the absence of Rapsyn [107]. Whether Lrp4, which associates with MuSK, is likewise concentrated at ‘synaptic’ sites in the absence of Rapsyn is not known.

Mutations in *Rapsyn* cause congenital myasthenia, and the most common mutation occurs at N88 in the third TPR repeat. The TPR repeats in Rapsyn, which can associate with one another, as well as bind Macf1, have been proposed to play a direct role in Rapsyn oligomerization and indirectly in AChR clustering [98,102]. Surprisingly, given that the TPR repeats are thought to regulate Rapsyn oligomerization, whereas the coiled-coil region is thought to mediate association with AChRs, mutation of N88 does not alter the ability of Rapsyn to self-cluster but instead impairs the ability of Rapsyn to cluster AChRs in 293 cells transfected with *Rapsyn* and *AChR* subunit cDNAs [108].

Rapsyn-mediated neddylation of AChRs, mediated by the RING finger in Rapsyn, plays an important role in clustering AChRs [101]. Addition of NEDD8 may be instructive for AChR clustering, possibly by serving as a recruitment motif for scaffolding proteins that anchor AChRs. Understanding how neddylation regulates AChR clustering, including Identification of proteins that may be recruited to neddylated AChRs, would be an important step forward.

Rapsyn is associated with AChRs in post-Golgi vesicles [109,110], so it is possible that Rapsyn has a role in directing transport vesicles to the postsynaptic membrane, as well as anchoring AChRs and other postsynaptic proteins in the plasma membrane. A further and better understanding of how Rapsyn targets and anchors postsynaptic proteins remains a burning and important question.

Rapsyn is associated with non-clustered AChRs, present on myotubes in the absence of Agrin stimulation and in extrasynaptic regions of denervated muscle, indicating that Rapsyn recruitment, on its own, is insufficient to stimulate AChR clustering [111]. Instead, these findings suggest that Agrin-stimulation alters Rapsyn or Rapsyn-associated proteins so that pre-bound Rapsyn now functions as an anchoring protein. The biochemical changes that follow MuSK stimulation and lead to Rapsyn clustering are not well understood.

Agrin stimulates tyrosine phosphorylation of the AChR β subunit, leading to recruitment of Rapsyn to the tyrosine phosphorylated β subunit and therefore additional Rapsyn bound to AChRs [96,111]. Preventing β subunit tyrosine phosphorylation inhibits this additional recruitment but does not block Agrin-stimulated clustering of AChRs, indicating that recruitment of Rapsyn to the tyrosine phosphorylated AChR β subunit is not essential to cluster AChRs [112]. Nonetheless, preventing AChR β subunit tyrosine phosphorylation leads to defects in development of postjunctional folds, a reduction in the density of synaptic AChRs and renders clustered AChRs more labile, indicating that this further recruitment of Rapsyn assists in stabilizing AChRs that are clustered at synapses or in muscle cells treated with Agrin [112]. Structural studies of AChR-rich electric organ membranes containing Rapsyn present a model that may explain how recruitment of Rapsyn to the tyrosine phosphorylated AChR β subunit may stabilize AChR clusters, as three unlike two molecules of Rapsyn are predicted to form an extensive cross-linked network of AChRs that would presumably stabilize AChRs [113].

## 3. Steps in Synapse Formation

### 3.1. Muscle Prepatterning

**Figure 2:**
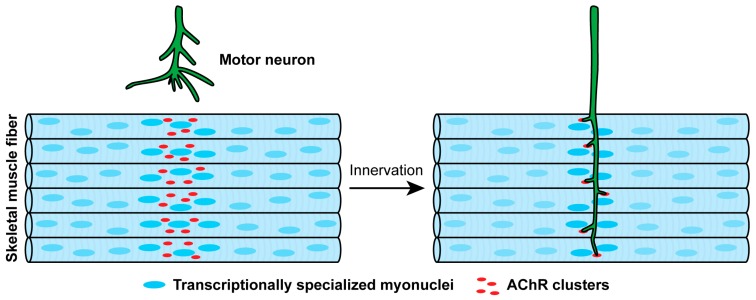
Steps in the formation of neuromuscular synapses.

Nearly twenty years ago it became clear that postsynaptic differentiation could be initiated in muscle in the absence of Agrin or indeed motor neurons [114,115,116,117,118]. These studies pointed to the ability of muscle to become pre-patterned or specialized in the prospective synaptic region in the absence of signals from motor neurons (Figure 2). Muscle pre-patterning, like synapse formation, requires MuSK. In zebrafish, MuSK is activated during pre-patterning by muscle-derived Wnts, which bind the Fz-like domain in MuSK [119]; in mice, MuSK is activated during pre-patterning by Lrp4 [57], which binds to the first Ig-like domain in MuSK [60]. The role of the MuSK Fz-like domain during synapse formation is controversial: in zebrafish, the Fz-like domain is dispensable for synapse formation [119], whereas conflicting results have been reported for the importance of the Fz-like domain in synapse formation in mice [120,121] (Figure 2).

During development, prior to and independent of innervation, AChRs are clustered in the prospective synaptic region of muscle. Innervation provides two signals that refine and sharpen this prepatterned arrangement so that AChRs become restricted to synaptic sites: ACh depolarizes muscle, which disperses AChR clusters, whereas Agrin activates MuSK, focally stabilizing AChRs in the muscle membrane that is in apposed to nerve terminals.

**Figure 3:**
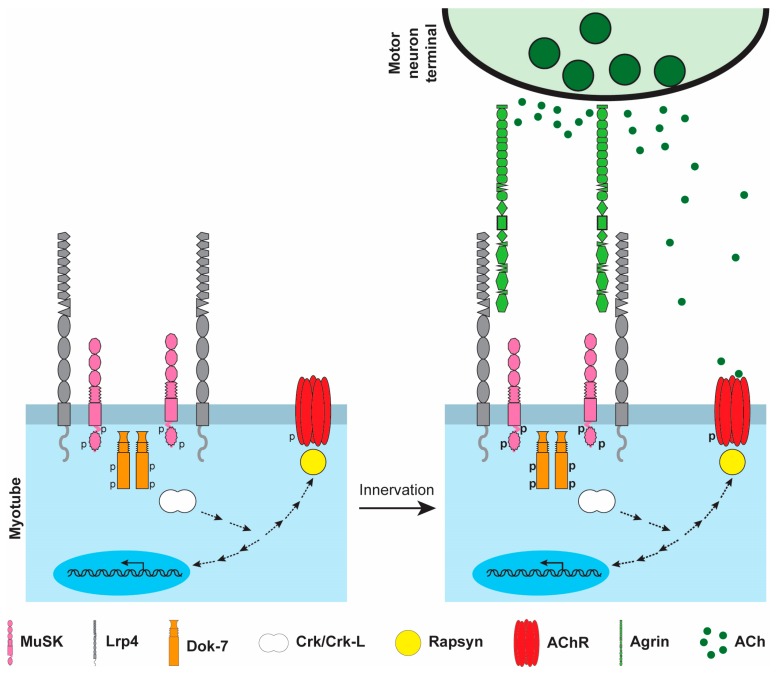
Mechanism for forming and maintaining neuromuscular synapses.

Muscle pre-patterning has a role in directing synapses to form in the central region of mammalian muscle. Once motor axons arrive in the muscle, usually entering near the center of the muscle, independent of MuSK [35,58], they branch from the main intramuscular nerve in a stereotyped manner, recognizing the pre-patterned region, and forming synapses adjacent to the main intramuscular nerve near the center of the muscle [58] (Figure 2). Motor neuron-derived Agrin then binds to Lrp4, potentiating the association between Lrp4 and MuSK and substantially increasing MuSK [39,59,60], thereby enhancing the pathways for anchoring postsynaptic proteins and synapse-specific transcription (Figure 3). As such, Agrin serves to form, consolidate and stabilize neuromuscular synapses.

Prepatterning requires Lrp4 and MuSK. Lrp4 binds to MuSK and stimulates MuSK phosphorylation, which is stabilized by Dok-7. Upon innervation, motor neuron-derived Agrin binds to Lrp4, further stimulating the interaction between Lrp4 and MuSK, increasing MuSK phosphorylation, and promoting downstream signaling.

### 3.2. Stabilizing and Dispersing AChR Clusters

The pre-patterned distribution of AChRs requires Lrp4 and MuSK [57,116,117,121]. Because Lrp4 binds to and stimulates MuSK, Lrp4 acts a cis-acting ligand for MuSK. Once activated by Lrp4, MuSK stimulates the clustering not only of AChRs but also of MuSK and Lrp4 [29,122]. Clustered Lrp4 then functions as a retrograde signal to motor neurons, causing motor axons to stop growing and to form specialized nerve terminals [71]. Agrin, released from nerve terminals, binds to Lrp4, stimulating an increase in association between Lrp4 and MuSK and a substantial increase in MuSK tyrosine phosphorylation [39,59,60]. As such, Lrp4 functions as a checkpoint at these three sequential steps in synapse formation, since each subsequent step cannot be executed without completion of the preceding Lrp4-dependent step.

Studies of the requirements for Agrin in muscle pre-patterning and innervation led to a hypothesis suggesting that innervation provides a second signal, in addition to Agrin, which assists in converting the pre-patterned distribution of AChR clusters to the more spatially restricted distribution of AChR clusters confined to synapses [116,117]. In this model, the Agrin/Lrp4/MuSK pathway acts to form and maintain postsynaptic differentiation, including AChR clustering, whereas the second nerve-derived signal, ACh, counteracts the clustering activity of Agrin by stimulating dispersal/removal of AChR clusters that are not opposed by nerve terminals and Agrin [123,124]. The mechanism by which ACh disperses AChR clusters is not well understood, but it appears that Calpain, a protease, as well as Cdk5, a serine/threonine kinase, play a role [123,125,126]. ACh treatment of cultured muscle cells increases the activities of Calpain and Cdk5, and the increase in Cdk5 activity is dependent upon Calpain. The ACh-mediated increase in Calpain/Cdk5 activity promotes dispersal of AChR clusters. In contrast, Agrin stimulates recruitment of Calpain to the TPR repeats in Rapsyn (see below), inhibiting Calpain activity. Much remains unknown: how does ACh stimulate Calpain activity; how does Rapsyn reduce Calpain activity; how does Agrin stimulate recruitment of Calpain to Rapsyn; does ACh alter the association between Calpain and Rapsyn; does Calpain have substrates other than p25/Cdk5, such as links between AChR/Rapsyn and a cytoskeleton, which could disperse AChR clusters? Moreover, because manipulating Calpain activity has a modest effect on dispersal of AChR clusters, additional pathways may act downstream from ACh to disassemble AChR clusters that are not contacted by nerve terminals and stabilized by Agrin.

### 3.3. Synapse Elimination and Maturation

During embryonic development, the single synaptic site on individual muscle fibers is innervated by approximately half dozen motor axons. Over the next several weeks, synapses are eliminated, so that each myofiber is ultimately innervated by a single motor axon. This rearrangement leads to a reduction in the number of muscle fibers innervated by each motor neuron (motor unit size) without a loss of motor neurons. The mechanisms responsible for this regulated pruning are not understood but appear to require competition among motor axon terminals, favoring the survival of the most active terminal at individual synapses [127]. Given the stabilizing role for Agrin/MuSK signaling, and the extinguishing role for ACh signaling during earlier steps in synapse formation, one wonders whether the same signaling system is employed later to regulate synapse elimination and editing.

Following these early steps in synapse formation, the structure and function of the neuromuscular synapse continues to mature [14,128]. Proteins that are required for the formation of synapses are invariably required for their maturation, but certain proteins, such as Src-family kinases, dystrobrevin and Neuregulin-1 play roles in the maturation and stabilization yet are not essential for the formation of neuromuscular synapses [91,129,130,131,132,133,134,135]. How these and other proteins regulate later steps in maturation is poorly understood.

### 3.4. Targets for Therapy to Maintain Neuromuscular Synapses and Reduce Denervation

Manipulation of the signaling mechanisms that are responsible for maintaining attachment of motor nerve terminals to muscle holds promise for neuromuscular and neurodegenerative diseases that are hallmarked by neuromuscular dysfunction and axon withdrawal [136,137,138,139].

In *SOD1 G93A* mice, a mouse model for amyotrophic lateral sclerosis (ALS), boosting retrograde signaling by modestly increasing *MuSK* gene expression decreases the rate and extent of denervation and improves motor performance [136]. Raising the levels of *Dok-7* gene expression in mice, either by adenoviral gene transfer or a *Dok-7* transgene stimulates MuSK phosphorylation and increases synaptic size [84]. In mouse models for congenital Dok-7 myasthenia, Emery-Dreifuss muscular dystrophy or ALS, a boost in *Dok-7* gene expression suppresses motor axon withdrawal, improves motor function and extends life span [137,139]. Although gene therapy in skeletal muscle remains a daunting approach, these findings suggest that similar approaches hold promise.

## 4. Conclusions and Discussion

Over the past two decades we have gained insight into the molecules and mechanisms by which motor neurons and myofibers communicate and instruct each other to form and maintain a synapse. Nevertheless, key aspects of these well-orchestrated interactions remain unclear. For example, Lrp4 is a critical retrograde signal for instructing motor axons to stop growing and to differentiate [71], but the presynaptic Lrp4 receptor and the mechanisms that mediate growth arrest and presynaptic differentiation are unknown. Moreover, because defects in the function of the Agrin/Lrp4/MuSK/Dok-7/Rapsyn pathway are responsible for neuromuscular disease, autoantibodies to the extracellular receptor of the Lrp4 receptor and mutations in this receptor may be responsible for myasthenia as well.

A loss of function in *Agrin*, *Lrp4*, *MuSK*, *Dok*-7, or *Rapsyn* results in a failure to form neuromuscular synapses, leading to respiratory failure and neonatal lethality. Despite the critical importance of these genes in synapse formation and survival, no other genes act in a redundant manner and serve as ‘back-up’ genes. AChRs are expressed in the postsynaptic membrane in 3- to 5-fold excess of that required for ACh to elicit an action potential and muscle contraction. Although this safety factor cannot overcome a complete loss of Agrin, Lrp4, MuSK, Dok-7 ,or Rapsyn function, this excess in AChR expression, particularly at the crests of the postjunctional folds directly across from active zones in the nerve terminal, may permit synapses to function well enough to allow for survival in instances when the activity of these essential synaptic proteins is reduced, possibly alleviating the precarious nature of single, non-redundant genes for an essential function.
